# PICN Nanocomposite as Dental CAD/CAM Block Comparable to Human Tooth in Terms of Hardness and Flexural Modulus

**DOI:** 10.3390/ma14051182

**Published:** 2021-03-03

**Authors:** Yohei Kawajiri, Hiroshi Ikeda, Yuki Nagamatsu, Chihiro Masaki, Ryuji Hosokawa, Hiroshi Shimizu

**Affiliations:** 1 Division of Oral Reconstruction and Rehabilitation, Department of Oral Functions, Kyushu Dental University, Fukuoka 803-8580, Japan; r16kawajiri@fa.kyu-dent.ac.jp (Y.K.); masaki@kyu-dent.ac.jp (C.M.); hosokawa@kyu-dent.ac.jp (R.H.); 2 Division of Biomaterials, Department of Oral Functions, Kyushu Dental University, Fukuoka 803-8580, Japan; yuki-naga@kyu-dent.ac.jp (Y.N.); r14shimizu@fa.kyu-dent.ac.jp (H.S.)

**Keywords:** CAD/CAM, polymer infiltrated ceramic network, nanocomposite, silica, restorative material, dental material, biomimetics, dental core, dental crown, mechanical properties

## Abstract

Polymer infiltrated ceramic network (PICN) composites are an increasingly popular dental restorative material that offer mechanical biocompatibility with human enamel. This study aimed to develop a novel PICN composite as a computer-aided design and computer-aided manufacturing (CAD/CAM) block for dental applications. Several PICN composites were prepared under varying conditions via the sintering of a green body prepared from a silica-containing precursor solution, followed by resin infiltration. The flexural strength of the PICN composite block (107.8–153.7 MPa) was similar to a commercial resin-based composite, while the Vickers hardness (204.8–299.2) and flexural modulus (13.0–22.2 GPa) were similar to human enamel and dentin, respectively. The shear bond strength and surface free energy of the composite were higher than those of the commercial resin composites. Scanning electron microscopy and energy dispersive X-ray spectroscopic analysis revealed that the microstructure of the composite consisted of a nanosized silica skeleton and infiltrated resin. The PICN nanocomposite block was successfully used to fabricate a dental crown and core via the CAD/CAM milling process.

## 1. Introduction

The fabrication of dental prostheses has shifted from conventional craftsmanship to digital techniques based on computer-aided design and computer-aided manufacturing (CAD/CAM) [1,2,3]. Specifically, recent advances in CAD/CAM technologies have allowed for the production of dental crowns, inlays, bridges and cores using block materials and the CAD/CAM milling process. In materials science, contemporary CAD/CAM blocks are categorized into three groups, namely metal-based (e.g., titanium alloy [4] and Co-Cr alloy [5]), ceramic-based (e.g., feldspathic porcelain [6], lithium disilicate glass [7] and zirconia [8]), or resin-based (e.g., acrylic resin [9] and resin composite (hereafter composite) [10]). An investigation of new composites already in use (e.g., poly(ether-ether-ketone) (PEEK) [11]) and some interesting research on new materials with hierarchized geometry [12] and biomechanical problems [13,14,15] (i.e., bruxism) have also been conducted thus far.

CAD/CAM blocks that offer excellent biocompatibility and mechanical properties in the oral environment have been practically implemented, but their mechanical properties differ from those of human tooth [16]. To overcome this issue, dental material development should consider biomimetics [17,18]. Biomimetic materials imitate a biological function and tissue morphology, where such dental materials have been previously investigated and reported [16,19,20,21]. Biomimetic dental restorative materials for prostheses should imitate the properties of natural tooth and its components, such as enamel and dentin [22]. Previous reports on the development of restorative materials that mimic tooth morphology and function [23,24] have demonstrated that highly biocompatible materials show promise as next-generation dental CAD/CAM blocks.

The realization of long-term tooth restoration using a dental material without fatal failure of the tooth or the restorative material is important. External stress tends to be concentrated at the interface of dissimilar materials with different mechanical properties [25]. The differences in the mechanical properties of a natural tooth, such as the hardness and elastic modulus, between the enamel and dentin are drastic. Further, the dentin–enamel junction, which is the gradient structure for connecting the enamel and dentin, moderates the stress concentration at the interface, thereby avoiding fatal failure of the natural tooth [26]. With regard to biomimetics, the mechanical properties of the restoration material and natural teeth should be the same. However, the Vickers hardness (H_V_) and the elastic modulus (E) of the recent CAD/CAM materials, such as zirconia (H_V_ = ca. 1300–1641, E = ca. 146–210 GPa [27]), lithium disilicate glass (H_V_ = ca. 580–676, E = ca. 95–96 GPa [27]), and resin-composites (H_V_ = ca. 65–98, E = ca. 9–15 GPa [10]), differ from those of dentin (H_V_ = ca. 20–90 [28], E = 16–25 GPa [29,30,31]) and enamel (H_V_ = ca. 270–420 [28], E = 48–105 GPa [32,33]).

The CAD/CAM material that offers mechanical properties that most closely mimic human enamel, thereby ensuring mechanical biocompatibility, is polymer infiltrated ceramic network (PICN) composite [34,35,36,37,38,39,40]. PICN composites have a dual network microstructure comprising a ceramic skeleton with infiltrated resin. This structure differs from conventional dispersed-filler (DF) composites, which comprise filler dispersed in a resin matrix [41]. PICN composite CAD/CAM blocks have been applied to indirect tooth restoration [42,43], where several basic and clinical studies have used a commercially available PICN composite named VITA ENAMIC, which comprises a silicate glass ceramic skeleton with infiltrated acrylic resin [34]. The previous studies have demonstrated that the PICN composites suitably mimic human enamel, specifically in terms of mechanical properties [16,44]. However, differences between the mechanical properties of PICN composites and teeth remain, thus there is room for further improvement.

This study aimed to develop a novel PICN composite CAD/CAM block material to mimic the mechanical properties of enamel and dentin. The PICN composite block was produced using a novel process.

## 2. Materials and Methods

The composition of the precursor solution was optimized to obtain a monolithic block without fatal cracks, and six PICN composites were prepared under different preparation conditions (sintering time, type of infiltration resin monomer, and polymerization schedule) (see Appendix A). The mechanical properties (flexural strength, flexural modulus, and Vickers hardness) of the PICN composite blocks were evaluated, and the bonding properties to resin cement were assessed based on shear bond strength (SBS) and surface free energy (SFE). Further, the microstructure of the PICN composite was determined using scanning electron microscopy (SEM). The resultant PICN composite block was used to produce a dental crown and core via CAD/CAM milling.

### 2.1. Materials

The regents used to produce the PICN composite are listed in Table 1. The resulting PICN composites were compared to the commercial composites (i.e., control samples) listed in Table 2.

### 2.2. Preparation of PICN Composite

**Figure 1:**
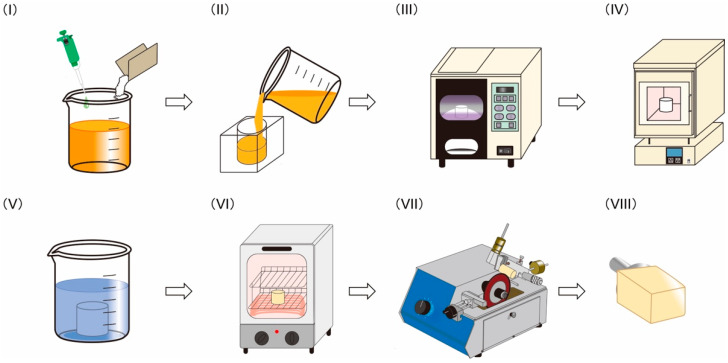
Fabrication of the polymer infiltrated ceramic network (PICN) composite to produce computer-aided design and computer-aided manufacturing (CAD/CAM) blocks: (I) preparation of light-curable precursor solution, (II) molding of precursor, (III) light-curing of precursor to form a green body, (IV) sintering of green body to form a porous body, (V) infiltration of resin monomer into the sintered porous body, (VI) heat-polymerization of the infiltrated body, and (VII) cutting of the PICN composite into (VIII) CAD/CAM blocks.

The PICN composites were produced using a novel process, as illustrated in Figure 1. This process included seven steps, as follows: (I) preparation of light-curable precursor solution, (II) molding of precursor, (III) light-curing of precursor to form a green body, (IV) sintering of green body to form a porous body, (V) infiltration of resin monomer into sintered porous body, (VI) heat-polymerization of the infiltrated body, and (VII) cutting the PICN composite to give CAD/CAM blocks. Six different PICN composites were produced by varying the preparation conditions, namely the sintering duration at 1150 °C, type of infiltrated resin monomer, and polymerization schedule for the infiltrated resin monomer.

The precursor solution (PS-1, see Appendix A and Table A1) were prepared with varying proportions of monomers (2-hydroxyethy methacrylate (HEMA) and triethylene glycol di-methacrylate (TEGDMA)) and solvents (2-phenoxyethanol (POE) and 1-propanol (PrOH)) with a fixed content of SiO_2_ nanoparticles and light initiator (phenylbis (2, 4, 6-trimethyl-benzoyl) phosphine oxide (BAPO). The reagents were mixed using a planetary centrifugal mixer (ARE-310, THINKY Corp., Tokyo, Japan) at 2000 rpm for 6 min, and defoamed for 1 min using the defoam mode of the mixer to remove microbubbles from the solution. The precursor solution was poured into transparent silicone mold (height = 20 mm; diameter = 18 mm) and light-cured using a light-irradiator (α-LIGHT II N, J. Morita Corp., Suita, Japan) for 10 min. The samples were dried in an oven at 80 °C for 1 week to fabricate a green body. The green bodies were sintered in a furnace according to the following heating schedule: heating from room temperature to 220 °C at 50 °C/h; isothermal hold at 220 °C for 6 h; heating to 600 °C at 100 °C/h; isothermal hold for 3 h; heating to 1150 °C at 100 °C/h; isothermal hold for 1, 2, or 3 h (Table 3); and cooling to room temperature inside the furnace. The sintered body was a porous silica block, which was immersed in a silane solution of ɤ-MPTS (0.5 g), ethanol (8.5 g), distilled water (1.0 g), and 1M HNO_3_ (100 μL) at room temperature for 3 h and dried in an oven (DY300, Yamato Scientific Co., Ltd., Tokyo, Japan) at 80 °C for 3 h. The silanized porous silica block was immersed in a resin monomer containing 0.5 wt% BPO at room temperature for 3 days. The monomer infiltrated silica block was heat-polymerized using the appropriate schedule for the monomer composition (Table 3) to give the PICN composite. The PICN composite was cut into blocks (12 × 15 × 10 mm^3^) to obtain CAD/CAM blocks.

### 2.3. Three-Point Bending Test

Each sample was cut and polished using emery papers up to #2000 to produce bar-shaped samples (width = 4 mm; length = 14 mm; thickness = 1.2 mm) (n = 10). The flexural strength and modulus of the samples were determined via three-point bending testing according to the standard procedure given in ISO 6872: 2008 [45]. A universal testing machine (AGS-H, Shimadzu Corp., Kyoto, Japan) with a support span of 12 mm and crosshead speed of 1 mm/min was used [10].

### 2.4. Vickers Hardness

After the three-point bending test, the fractured samples were used for the measurement of Vickers hardness according to the standard procedure given in ISO 6872: 2008 [45]. A hardness tester (HMV-G21ST, Shimadzu Corp., Kyoto, Japan) with a load of 200 g and dwell time of 15 s was used (n = 10) [39].

### 2.5. Inorganic Content

After hardness testing, the samples were weighed using an electric balance and calcined at 600 °C for 3 h in air to remove all organic matter. According to the literature [46], the organic matter in the sample, such as poly-UDMA and poly-TEGDMA, would be completely combusted at that temperature. The residue after calcination was weighed, and the inorganic content of the sample was calculated as the difference between the specimen weight before and after calcination (n = 10).

### 2.6. Shear Bond Strength

The SBS between the samples and a commercial resin cement was measured using a conventional procedure [47]. Disk-shaped samples (diameter = 10 mm, thickness = 1.5 mm) (n = 20) were polished using emery papers up to #1000. Silane primer (Porcelain primer, SHOFU Inc., Kyoto, Japan) was applied on the sample surface, and the resin cement (Resicem, SHOFU Inc., Kyoto, Japan) was loaded on the sample surface and cured using the light irradiator for 5 min. The cement-cured sample was held under ambient conditions for 60 min, and stored in distilled water at 37 °C for 24 h. The samples were divided into two groups to establish the properties before and after thermocycling, denoted as the 0-thermocycle and 20,000-thermocycle groups, respectively. Thermocycling was conducted by alternately immersing the samples in water baths at 5 and 55 °C for 20,000 cycles of 60 s in each bath. SBS testing of the 0-thermocycle and 20,000-thermocycle group samples was performed using the universal testing machine (n = 10). After SBS testing, the cement-debonded surface was observed using an optical microscope to classify the failure modes as one of three types, namely adhesive failure at the cement–sample interface, cohesive failure within the sample, or mixed adhesive and cohesive failure.

### 2.7. Surface Free Energy

The SFE of the samples (n = 10) was determined based on the contact angles between the sample surface and two liquids, namely distilled water and diiodomethane (Kanto Chemical Co., Inc. Tokyo, Japan). A contact angle meter (DMe-211, Kyowa Interface Science Co., Ltd., Saitama, Japan) was used under ambient conditions at 20 ± 3 °C (n = 10). The SFE was calculated using the Owens–Wendt theory [48] as follows:

(1) $$\sqrt{\gamma_{L1}^{d}\gamma_{S}^{d}}+\sqrt{\gamma_{L1}^{P}+\gamma_{s}^{p}}=\frac{\gamma_{L1}^{total}\left(1+\cos\theta_{L1}\right)}{2},$$

(2) $$\sqrt{\gamma_{L2}^{d}\gamma_{S}^{d}}+\sqrt{\gamma_{L2}^{P}+\gamma_{s}^{p}}=\frac{\gamma_{L2}^{total}\left(1+\cos\theta_{L2}\right)}{2},$$

(3) $$\sqrt{\gamma^{total}=\gamma^{d}+\gamma^{p}}$$

where *θ* denotes the contact angle for the liquids, the subscript indices *L*1 and *L*2 indicate water and diiodomethane, respectively, and *γ^total^*, *γ^p^*, and *γ^d^* are the total SFE, polar (hydrogen) SFE component, and dispersive SFE component of the sample, respectively. The SFE values for water and diiodomethane were based on previously reported values [48].

### 2.8. Microstructural Analysis

SEM and elemental mapping images of the samples were acquired using SEM (JCM-6000Plus NeoScope, JEOL Ltd., Tokyo, Japan) equipped with an energy dispersive X-ray spectroscopy (EDX) spectrometer.

### 2.9. CAD/CAM Milling of PICN Composite Block

The PICN composite block was milled to form a dental crown (maxillary right first premolar) (n = 1) and dental core (maxillary right first premolar) (n = 1) using a commercial CAD/CAM system (inLab MC X5, Dentsply Sirona Inc., Charlotte, NC, USA).

### 2.10. Statistical Analysis

Statistical analysis was performed using EZR software (Saitama Medical Center, Jichi Medical University, Saitama, Japan). Analysis of the flexural strength, flexural modulus, Vickers hardness, SBS and SFE was conducted using one-way analysis of variance (ANOVA) for multiple comparisons in the groups. Tukey’s post hoc test was performed for the statistically significant groups. A significance level (*p*) of 0.05 was used for all analyses.

## 3. Results

### 3.1. Mechanical Properties

The mechanical properties and inorganic contents of the PICN composites and commercial composites are given in Table 4. The flexural strength of the PICN composites was influenced by the preparation conditions, namely sintering time, infiltrated resin monomer, and polymerization schedule, where the highest flexural strength (153.7 MPa) was achieved in sample 2h-U-60. Further, the flexural modulus and Vickers hardness of the PICN composites increased with sintering time. The inorganic content of the PICN composites increased with increasing the sintering time from 71.2 wt% to 89.6 wt%. The 2h-U-60 composite was chosen as the representative PICN composite for the subsequent steps, including SBS analysis, SFE analysis, SEM-EDX analysis, and CAD/CAM milling fabrication.

### 3.2. Shear Bond Strength

**Figure 2:**
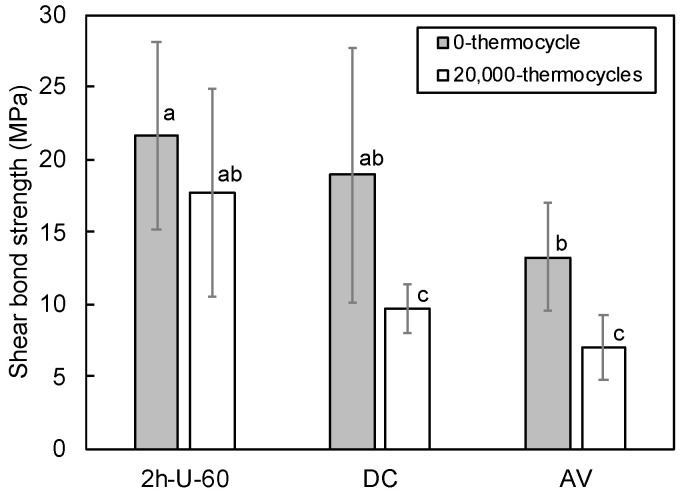
Shear bond strength of the PICN composite (2h-U-60) and commercial composites (DC and AV) at 0 and 20,000 thermocycles. Different letters indicate a significant difference between the groups (*p* < 0.05, Tukey test, n = 10), and the vertical bars denote standard deviation.

The SBS test results of the PICN composite (2h-U-60) and commercial composites (DC and AV) before and after 20,000 thermocycles are given in Figure 2. Before thermocycling groups, there was difference between the PICN composite and AV. After thermocycling, the SBS of the PICN composite was significantly higher than those of DC and AV. Further, there was no significant change in the SBS value of the PICN composite between before and after thermocycling, while the SBSs of DC and AV significantly decreased.

**Figure 3:**
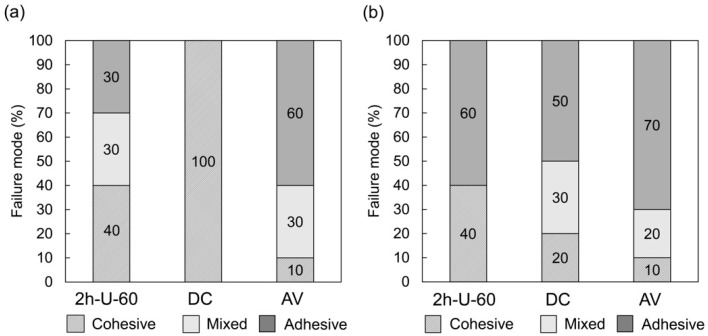
Failure modes of the PICN composite (2h-U-60) and commercial composites (DC and AV) after shear bond strength testing at (**a**) 0 and (**b**) 20,000 thermocycles (n = 10).

AV exhibited the fewest cohesive failures before thermocycling, followed by the PICN composite and then DC (Figure 3). After thermocycling, AV exhibited the fewest, followed by DC and PICN composite. There was no difference in the incidence of cohesive failure of the PICN composite before and after thermocycling.

### 3.3. Surface Free Energy

**Figure 4:**
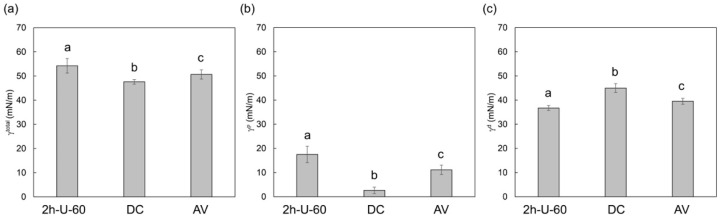
SFE of the PICN composite (2h-U-60) and commercial composites (DC and AV) given as (**a**) total SFE (*γ^total^*); (**b**) polar component of SFE (*γ^p^*); (**c**) dispersive component of SFE (*γ^d^*). Different letters indicate a significant difference between the groups (*p* < 0.05, Tukey test, n = 10), and the vertical bars denote standard deviation.

The PICN composite (2h-U-60) exhibited a higher total SFE (Figure 4a) and polar SFE component (Figure 4b) than the commercial composites (DC and AV), as well as the lowest dispersive SFE component (Figure 4c).

### 3.4. Microstructure

**Figure 5:**
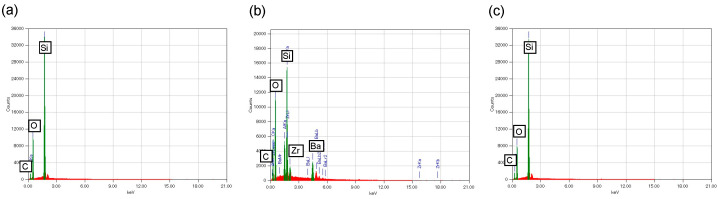
Energy dispersive X-ray spectroscopy (EDX) spectra of (**a**) PICN composite (2h-U-60); (**b**) DC commercial composite; and (**c**) AV commercial composite.

The EDX spectra of the PICN composite (2h-U-60) was compared to those of the commercial composites (DC and AV) (Figure 5). The PICN composite exhibited peaks attributed to silicon and oxygen, which corresponded to the silica skeleton, as well as a carbon peak due to the infiltrated resin. AV exhibited silicon and oxygen peaks related to its silica fillers, and carbon peaks due to the resin matrix, while DC exhibited peaks attributed to silicon, oxygen and carbon, as well as aluminum, barium, zirconium due to the barium glass and zirconia fillers.

**Figure 6:**
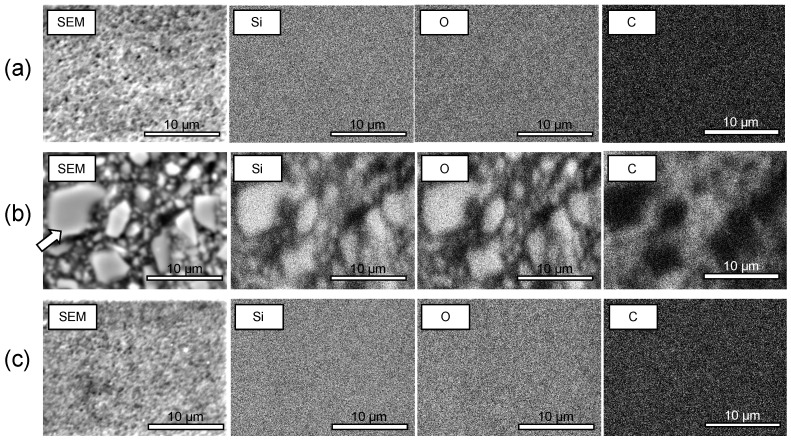
SEM images and EDX elemental mapping images of silicon (Si), oxygen (O), and carbon (C) of (**a**) PICN composite (2h-U-60); (**b**) DC commercial composite; and (**c**) AV commercial composite. The white arrow in (Figure **c**) indicates the filler. The silica skeleton (Figure **a**) and the silica nanoparticles (Figure **c**) were homogeneous in nanoscale.

SEM and EDX elemental mapping images were acquired to evaluate the silica (SiO_2_) inorganic component (oxygen and silicon) and the resin component (carbon) (Figure 6). The PICN composite exhibited a uniform PICN nanostructure, while DC and AV comprised nano- and in microsized dispersed-filler structures, respectively.

### 3.5. CAD/CAM Milling

**Figure 7:**
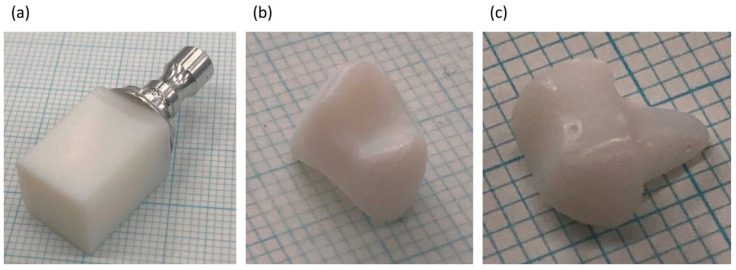
Digital photographs of the PICN composite (2h-U-60) (**a**) CAD/CAM block; (**b**) dental crown (maxillary right first premolar); and (**c**) dental core (maxillary right first premolar).

The PICN composite was used to produce a CAD/CAM block, which was milled to give a dental crown and dental core (Figure 7). The prepared PICN composite monolith block did not exhibit any cracks, while the milled crown and core exhibited no fatal damage such as edge chipping.

## 4. Discussion

The effect of the PICN composite preparation conditions on the mechanical properties was evaluated (Table 4). The infiltrated resin monomer affected the flexural strength, where the addition of UDMA (2h-U-60; 153.7 MPa) significantly enhanced the flexural strength compared to the composite prepared with only TEGDMA (2h-T-60; 117.7 MPa). TEGDMA has a lower strength than UDMA, and is usually used to dilute UDMA [49,50], which led to the superior flexural strength of the UDMA-infiltrated samples compared to the TEGDMA-infiltrated samples. The flexural strength was also affected by polymerization schedule, and was significantly higher in the sample polymerized at 60 °C for 5 days followed by 80 °C for 1 day (2h-U-60; 153.7 MPa) compared that polymerized at 100 °C for 1 day (2h-U-100; 119.0 MPa). Polymerization led to volume shrinkage, which typically generates internal stress within the sample [51]. Slower polymerization moderated internal stress in the sample [52], thus the internal stress during polymerization of the infiltrated monomer resin in the 2h-U-60 sample was less than that of the 2h-U-100 sample. Sintering time affected both the Vickers hardness and flexural modulus of the PICN composite, which increased with increasing sintering time in 1h-U-60, 2h-U-60, and 3h-U-60. Sintering of the silica particles progressed over time, which led to a stronger silica skeleton after a longer sintering time. This phenomenon was supported by the increase in inorganic (silica) content of the sample from 73.2 wt% for 1 h sintering (1h-U-60) to 89.6 wt% for 3 h (3h-U-60).

Vickers hardness and flexural modulus are import mechanical properties in dental restorative materials, where the Vickers hardness of the PICN composites (200.8–299.2) was significantly higher than those of the commercial composites (82.7 for DC and 72.5 for AV). This hardness is closer to that of enamel (270–420 [28]) rather than dentin (20–90 [28]), where the 3h-U-60 sample exhibited a particularly compatible hardness with enamel. The flexural modulus of the PICN composites (13.0–22.2 GPa) was also higher than those of the commercial composites (8.3 for DC and 11.8 for AV). These values were more similar to those of dentin (16–25 GPa [29,30,31]) compared to enamel (48–105 GPa [32,33]). Overall, the PICN composite was mechanically biocompatible with the hardness of enamel and flexural (elastic) modulus of dentin. The mechanical properties of the proposed PICN composite emulates the Vickers hardness and elastic modulus of enamel more closely than dentin, unlike previously reported PICN composites [34,38,53].

The superior SBS of the PICN composite with the resin cement compared to the commercial composites (DC and AV) led to the PICN composite undergoing cohesive failure after thermocycling more often than the other composites. This was attributed to the preferable bond durability between the PICN composite and resin cement, which was related to its surface properties. The SFE analysis revealed that the polar SFE component and total SFE of the PICN composite were significantly higher than those of commercial composites. A previous study [54] demonstrated that the large polar SFE component of this type of composite is indicative of a large number of surface silanol groups, where the active site of the silane coupling agent allowed for higher bond strength to the resin cement. This facilitated effective bonding between the resin cement (with silane primer) and the PICN composite.

The microstructure of the PICN was too fine for observation using SEM-EDX analysis (Figure 6). This demonstrated that the structure of the proposed PICN composite comprised a nanoscale silica skeleton with infiltrated resin. Thus, the proposed nanocomposite had a finer ceramic skeleton than previously reported microscale PICN composites [34,36,37].

To demonstrate the possible fabrication of a dental crown or core using the prepared PICN nanocomposite block, we attempted to mill the PICN nanocomposite block using the commercial CAD/CAM milling system. The PICN composite CAD/CAM block was successfully milled to form a dental crown and core without fatal damages (Figure 7).

Within the limitation of this study, the Vickers hardness and elastic modulus of the PICN nanocomposite block are comparable to those of enamel and dentin. These findings suggest the application potential of the proposed PICN nanocomposite as a biomimetic dental restorative material. The presented PICN nanocomposite clearly exhibited comparable Vickers hardness and lower elastic modulus than those of the alkali-aluminosilicate-glass skeleton (e.g., VITA ENAMIC; H_V_ = ca. 177–190, E = ca. 29–38 GPa [10,34] or zirconia skeleton (H_V_ = ca. 300, E = ca. 44 GPa [55]). Thus, the elastic modulus of the presented PICN nanocomposite is relatively similar to that of dentin. This can be ascribed to the microstructure of the presented PICN nanocomposite because the ceramic skeleton is consistent with the nanosized silica. The restorative material (e.g., a crown) developed using the presented PICN nanocomposite may overcome the problems caused by the difference in hardness between the opposite tooth and restorative material and by the difference in elastic modulus between the abutment tooth and restorative material. In the future, the wear and fatigue behaviors of the PICN nanocomposite are expected to be studied. In addition, in vivo studies will be conducted to compare the mechanical behaviors of such materials with those of conventional restorative materials.

## 5. Conclusions

A monolithic PICN nanocomposite block comprising a silica skeleton and infiltrated UDMA-based resin was prepared by optimizing the processing conditions. The PICN nanocomposite exhibited a similar Vickers hardness to enamel and flexural modulus to dentin, as well as excellent bond properties with resin cement. The PICN nanocomposite block was used to form a biomimetic dental crown and core via CAD/CAM milling. The proposed PICN nanocomposite shows great promise as a mechanically biocompatible restorative material.

## Figures and Tables

**Table 1:** Reagents used for preparation of PICN composites.

Acronym	Material Type	Manufacturer	Product Name	Purity (%)
Silica	Nanoparticles	NiPPON AEROSIL, Tokyo, Japan	OX50	99.8
HEMA	Monomer	FujiFilm Wako Chemical, Osaka, Japan	2-hydroxyethy methacrylate	95.0
TEGDMA	Monomer	FujiFilm Wako Chemical, Osaka, Japan	Triethylene glycol dimethacrylate	90.0
POE	Solvent	FujiFilm Wako Chemical, Osaka, Japan	2-phenoxyethanol	99.0
PrOH	Solvent	FujiFilm Wako Chemical, Osaka, Japan	1-propanol	99.5
BAPO	Light-initiator	FujiFilm Wako Chemical, Osaka, Japan	Phenylbis (2, 4, 6-trimethyl-benzoyl) phosphine oxide	97.0
ɤ-MPTS	Silane coupling agent	Shin-Etsu Chemical, Tokyo, Japan	3-methacryl oxypropyl trimethoxysilane	99.9
UDMA	Monomer	Sigma-Aldrich, St. Louis, MO, USA	Urethane dimethacrylate	97.0
BPO	Heat-initiator	Alfa Aesar, Lancashire, UK	Benzoyl peroxide	97.0

**Table 2:** Commercial resin composite control samples.

Acronym	Material Type	Product	Manufacturer	Monomer Composition	Filler Composition
DC*	Direct resin composite	Clear fill DC core Auto Mix ONE	Kuraray Noritake Dentall, Tokyo, Japan	Bis-GMA, methacrylic monomer, TEGDMA, other	Silica, Alumina, Silica-based glass
AV	Indirect resin composite (CAD/CAM block)	KATANA AVENCIA Block	Kuraray Noritake Dentall, Tokyo, Japan	UDMA, methacrylic monomer, other	Silica, Aulmina

* The specimen was formed via a light-curing by following manufacture’s instructions and used for the experiment.

**Table 3:** Preparation conditions for the PICN composites (sintering time at 1150 °C, infiltrated resin monomer, and polymerization schedule).

Sample Name	Sintering Time	Monomer	Polymerization Schedule
2h-T-100	2 h	TEGDMA *	100 °C 1d ***
2h-T-60	2 h	TEGDMA *	60 °C 5d → 80 °C 1d ****
2h-U-100	2 h	UDMA+TEGDMA **	100 °C 1d ***
1h-U-60	1 h	UDMA+TEGDMA **	60 °C 5d → 80 °C 1d ****
2h-U-60	2 h	UDMA+TEGDMA **	60 °C 5d → 80 °C 1d ****
3h-U-60	3 h	UDMA+TEGDMA **	60 °C 5d → 80 °C 1d ****

* Infiltrated resin monomer is TEGDMA only. ** Infiltrated resin monomer is a mixture of UDMA and TEGDMA (4:1 weight ratio). *** Infiltrated resin was heat-polymerized at 100 °C for 1 day. **** Infiltrated resin was heat-polymerized at 60 °C for 5 days and at 80 °C for 1 day.

**Table 4:** Mechanical properties and inorganic content of the PICN composites and commercial composites (DC and AV) given as mean values (with standard deviation). Different letters indicate a significant difference between the groups (*p* < 0.05, Tukey test, n = 10).

Sample Name	Flexural Strength (MPa)	Flexural Modulus (GPa)	Vickers Hardness	Inorganic Content (wt%)
2h-T-100	107.8 (8.0) a	13.4 (1.3) a	204.8 (12.8) a	71.8 (3.1) a
2h-T-60	117.6 (6.5) a	13.0 (1.1) a	200.8 (13.0) a	71.2 (3.3) a
2h-U-100	119.0 (13.6) a	13.5 (1.6) a	210.3 (10.1) a	73.0 (3.4) a
1h-U-60	130.8 (19.2) ab	14.3 (1.9) a	213.6 (13.7) a	73.2 (2.9) a
2h-U-60	153.7 (9.6) b	16.9 (2.0) ab	218.3 (16.9) a	75.6 (3.3) a
3h-U-60	129.9 (25.2) ab	22.2 (3.6) c	299.2 (30.1) b	89.6 (5.6) b
DC	143.4 (11.5) b	8.3 (0.9) d	82.7 (7.02) c	69.4 (0.9) a
AV	208.0 (24.8) c	11.8 (2.2) a	72.5 (7.16) c	60.6 (1.5) c

## Data Availability

The date presented in this study are available on request from the corresponding author.

## Appendix A

### Optimization of Precursor Solution

Six precursor solutions, referred to as PS-1 to PS-6, were prepared with varying proportions of monomers (HEMA and TEGDMA) and solvents (POE and PrOH) with a fixed content of SiO_2_ nanoparticles and light initiator (BAPO) (supplemental Table). The reagents were mixed using the planetary centrifugal mixer. Monolithic porous silica blocks with a cylindrical shape (height = 20 mm; diameter = 18 mm) were formed using the precursor solutions via sintering. The green bodies were sintered in a furnace according to the following heating schedule: heating from room temperature to 220 °C at 50 °C/h; isothermal hold at 220 °C for 6 h; heating to 600 °C at 100 °C/h; isothermal hold for 3 h; heating to 1150 °C at 100 °C/h; isothermal hold for 2 h; and cooling to room temperature inside the furnace.

The monolithic porous silica blocks produced using precursor solutions PS-2, PS-3, PS-4, PS-5 and PS-6 formed fatal cracks during the sintering due to shrinkage stress. However, the monolithic porous silica block formed using PS-1 exhibited no cracks despite shrinking during the sintering process. Thus, PS-1 was used further in the present study, and the resulting monolithic porous silica blocks were successfully used to fabricate monolithic PICN composite blocks via the subsequent infiltration and polymerization processing steps.

Crack generation is a complicated phenomenon, and the mechanism through which cracking was suppressed in the PS-1 PICN composite has not yet been clarified. However, it is speculated that the appropriate ratio of resin monomers (HEMA and TEGDMA) and solvents (POE and PrOH) provided sufficient mechanical strength within the green body during light curing, which allowed for the structure to overcome the shrinkage stress generated during the subsequent sintering step. A PICN composite CAD/CAM block material must be capable of forming a monolithic block without fatal cracks. However, typical PICN composites tend to crack due to shrinkage during the sintering process. Therefore, determination of the optimal precursor solution composition was a critical step to ensure that monolithic blocks without fatal cracks were produced.

**Table A1:** Composition (g) of the precursor solutions.

Precursor Solution	Monomer		Solvent		Nanoparticles	Initiator
	HEMA	TEGDMA	POE	PrOH	Silica	BAPO
PS-1	8.0	0.8	1.8	7.0	22.0	0.4
PS-2	16.0	1.6	0	0	22.0	0.4
PS-3	8.8	0	1.8	7.0	22.0	0.4
PS-4	0	8.8	1.8	7.0	22.0	0.4
PS-5	8.0	0.8	8.8	0	22.0	0.4
PS-6	8.0	0.8	0	8.8	22.0	0.4

## References

[1] Alghazzawi T.F. (2016). Advancements in CAD/CAM technology: Options for practical implementation. J. Prosthodont. Res..

[2] Spitznagel F.A., Boldt J., Gierthmuehlen P.C. (2018). CAD/CAM ceramic restorative materials for natural teeth. J. Dent. Res..

[3] Yamaguchi S., Lee C., Karaer O., Ban S., Mine A., Imazato S. (2019). Predicting the debonding of CAD/CAM composite resin crowns with AI. J. Dent. Res..

[4] Yilmaz B., Alshahrani F.A., Kale E., Johnston W.M. (2018). Effect of feldspathic porcelain layering on the marginal fit of zirconia and titanium complete-arch fixed implant-supported frameworks. J. Prosthet. Dent..

[5] Kim H.R., Jang S.H., Kim Y.K., Son J.S., Min B.K., Kim K.H., Kwon T.Y. (2016). Microstructures and mechanical properties of Co-Cr dental alloys fabricated by three CAD/CAM-based processing techniques. Materials.

[6] Blackburn C., Rask H., Awada A. (2018). Mechanical properties of resin-ceramic CAD-CAM materials after accelerated aging. J. Prosthet. Dent..

[7] Lawson N.C., Bansal R., Burgess J.O. (2016). Wear, strength, modulus and hardness of CAD/CAM restorative materials. Dent. Mater..

[8] Ban S. (2020). Chemical durability of high translucent dental zirconia. Dent. Mater. J..

[9] McLaughlin J.B., Ramos V.J., Dickinson D.P. (2019). Comparison of fit of dentures fabricated by traditional techniques versus CAD/CAM technology. J. Prosthodont..

[10] Lauvahutanon S., Takahashi H., Shiozawa M., Iwasaki N., Asakawa Y., Oki M., Finger W.J., Arksornnukit M. (2014). Mechanical properties of composite resin blocks for CAD/CAM. Dent. Mater. J..

[11] Souza J.C.M., Correia M.S.T., Oliveira M.N., Silva F.S., Henriques B., Novaes de Oliveira A.P., Gomes J.R. (2020). PEEK-matrix composites containing different content of natural silica fibers or particulate lithium-zirconium silicate glass fillers: Coefficient of friction and wear volume measurements. Biotribology.

[12] Nakonieczny D.S., Antonowicz M., Paszenda Z.K. (2020). Cenospheres and their application advantages in biomedical engineering—A systematic review. Rev. Adv. Mater. Sci..

[13] Faus-Matoses V., Ruiz-Bell E., Faus-Matoses I., Ozcan M., Salvatore S., Faus-Llacer V.J. (2020). An 8-year prospective clinical investigation on the survival rate of feldspathic veneers: Influence of occlusal splint in patients with bruxism. J. Dent..

[14] Nakonieczny D.S., Marcin B., Sambok A., Antonowicz M., Paszenda Z.K., Ziębowicz A., Krawczyk C., Ziębowicz B., Lemcke H., Kałużyński P. (2019). Ageing of zirconia dedicated to dental prostheses for bruxers part 1: Influence of accelerating ageing for surface topography and mechanical properties. Rev. Adv. Mater. Sci..

[15] D’Addazio G., Santilli M., Rollo M.L., Cardelli P., Rexhepi I., Murmura G., Al-Haj Husain N., Sinjari B., Traini T., Ozcan M. (2020). Fracture resistance of zirconia-reinforced lithium silicate ceramic crowns cemented with conventional or adhesive systems: An in vitro study. Materials.

[16] Eldafrawy M., Nguyen J.F., Mainjot A.K., Sadoun M.J. (2018). A functionally graded PICN material for biomimetic CAD-CAM blocks. J. Dent. Res..

[17] Ritchie R.O. (2011). The conflicts between strength and toughness. Nat. Mater..

[18] Wilmers J., Bargmann S. (2020). Nature’s design solutions in dental enamel: Uniting high strength and extreme damage resistance. Acta Biomater..

[19] Du J., Niu X., Rahbar N., Soboyejo W. (2013). Bio-inspired dental multilayers: Effects of layer architecture on the contact-induced deformation. Acta Biomater..

[20] Madfa A.A., Yue X.G. (2016). Dental prostheses mimic the natural enamel behavior under functional loading: A review article. Jpn. Dent. Sci. Rev..

[21] Al-Jawoosh S., Ireland A., Su B. (2018). Fabrication and characterisation of a novel biomimetic anisotropic ceramic/polymer-infiltrated composite material. Dent. Mater..

[22] Zafar M.S., Amin F., Fareed M.A., Ghabbani H., Riaz S., Khurshid Z., Kumar N. (2020). Biomimetic aspects of restorative dentistry biomaterials. Biomimetics.

[23] Petrini M., Ferrante M., Su B. (2013). Fabrication and characterization of biomimetic ceramic/polymer composite materials for dental restoration. Dent. Mater..

[24] Oshima M., Inoue K., Nakajima K., Tachikawa T., Yamazaki H., Isobe T., Sugawara A., Ogawa M., Tanaka C., Saito M. (2014). Functional tooth restoration by next-generation bio-hybrid implant as a bio-hybrid artificial organ replacement therapy. Sci. Rep..

[25] Kim J.W., Bhowmick S., Chai H., Lawn B.R. (2007). Role of substrate material in failure of crown-like layer structures. J. Biomed. Mater. Res. Part B.

[26] Imbeni V., Kruzic J., Marshall G., Marshall S., Ritchie R. (2005). The dentin–enamel junction and the fracture of human teeth. Nat. Mater..

[27] Homaei E., Farhangdoost K., Tsoi J.K.H., Matinlinna J.P., Pow E.H.N. (2016). Static and fatigue mechanical behavior of three dental CAD/CAM ceramics. J. Mech. Behav. Biomed. Mater..

[28] Warkentin M., Freyse C., Specht O., Behrend D., Maletz R., Janda R., Ottl P. (2018). Correlation of ultrasound microscopy and Vickers hardness measurements of human dentin and enamel—A pilot study. Dent. Mater..

[29] Xu H.H., Smith D.T., Jahanmir S., Romberg E., Kelly J.R., Thompson V.P., Rekow E.D. (1998). Indentation damage and mechanical properties of human enamel and dentin. J. Dent. Res..

[30] Kinney J.H., Balooch M., Marshall G.W., Marshall S.J. (1999). A micromechanics model of the elastic properties of human dentine. Arch. Oral. Biol..

[31] Fong H., Sarikaya M., White S., Snead M. (1999). Nano-mechanical properties profiles across dentin–enamel junction of human incisor teeth. Mater. Sci. Eng. C.

[32] Ausiello P., Rengo S., Davidson C.L., Watts D.C. (2004). Stress distributions in adhesively cemented ceramic and resin-composite Class II inlay restorations: A 3D-FEA study. Dent. Mater..

[33] He L.H., Swain M.V. (2007). Nanoindentation derived stress-strain properties of dental materials. Dent. Mater..

[34] Della Bona A., Corazza P.H., Zhang Y. (2014). Characterization of a polymer-infiltrated ceramic-network material. Dent. Mater..

[35] Nguyen J.F., Ruse D., Phan A.C., Sadoun M.J. (2014). High-temperature-pressure polymerized resin-infiltrated ceramic networks. J. Dent. Res..

[36] El Zhawi H., Kaizer M.R., Chughtai A., Moraes R.R., Zhang Y. (2016). Polymer infiltrated ceramic network structures for resistance to fatigue fracture and wear. Dent. Mater..

[37] He L.H., Swain M. (2011). A novel polymer infiltrated ceramic dental material. Dent. Mater..

[38] Li J., Cui B.C., Lin Y.H., Deng X.L., Li M., Nan C.W. (2016). High strength and toughness in chromatic polymer-infiltrated zirconia ceramics. Dent. Mater..

[39] Ikeda H., Nagamatsu Y., Shimizu H. (2019). Preparation of silica-poly (methyl methacrylate) composite with a nanoscale dual-network structure and hardness comparable to human enamel. Dent. Mater..

[40] Facenda J.C., Borba M., Corazza P.H. (2018). A literature review on the new polymer-infiltrated ceramic-network material (PICN). J. Esthet. Restor. Dent..

[41] Mainjot A.K., Dupont N.M., Oudkerk J.C., Dewael T.Y., Sadoun M.J. (2016). From artisanal to CAD-CAM blocks: State of the art of indirect composites. J. Dent. Res..

[42] Goujat A., Abouelleil H., Colon P., Jeannin C., Pradelle N., Seux D., Grosgogeat B. (2018). Mechanical properties and internal fit of 4 CAD-CAM block materials. J. Prosthet. Dent..

[43] Conejo J., Ozer F., Mante F., Atria P.J., Blatz M.B. (2020). Effect of surface treatment and cleaning on the bond strength to polymer-infiltrated ceramic network CAD-CAM material. J. Prosthet. Dent..

[44] Ludovichetti F.S., Trindade F.Z., Werner A., Kleverlaan C.J., Fonseca R.G. (2018). Wear resistance and abrasiveness of CAD-CAM monolithic materials. J. Prosthet. Dent..

[45] ISO (2008). ISO 6872. Dentistry—Ceramic Materials.

[46] Alarcon R.T., Gaglieri C., Bannach G. (2018). Dimethacrylate polymers with different glycerol content. J. Therm. Anal. Calorim..

[47] Yano H.T., Ikeda H., Nagamatsu Y., Masaki C., Hosokawa R., Shimizu H. (2020). Effects of alumina airborne-particle abrasion on the surface properties of CAD/CAM composites and bond strength to resin cement. Dent. Mater. J..

[48] Owens D.K., Wendt D.T. (1969). Estimation of the surface free energy of polymers. J. Appl. Polym. Sci..

[49] Floyd C.J., Dickens S.H. (2006). Network structure of Bis-GMA-and UDMA-based resin systems. Dent. Mater..

[50] Lin C.H., Lin Y.M., Lai Y.L., Lee S.Y. (2020). Mechanical properties, accuracy, and cytotoxicity of UV-polymerized 3D printing resins composed of Bis-EMA, UDMA, and TEGDMA. J. Prosthet. Dent..

[51] Ferracane J.L. (2005). Developing a more complete understanding of stresses produced in dental composites during polymerization. Dent. Mater..

[52] Gad M.M., Fouda S.M., ArRejaie A.S., Al-Thobity A.M. (2019). Comparative effect of different polymerization techniques on the flexural and surface properties of acrylic denture bases. J. Prosthodont..

[53] Kang L., Zhou Y., Lan J., Yu Y., Cai Q., Yang X. (2020). Effect of resin composition on performance of polymer-infiltrated feldspar-network composites for dental restoration. Dent. Mater. J..

[54] Yano H.T., Ikeda H., Nagamatsu Y., Masaki C., Hosokawa R., Shimizu H. (2020). Correlation between microstructure of CAD/CAM composites and the silanization effect on adhesive bonding. J. Mech. Behav. Biomed. Mater..

[55] Li K., Kou H., Rao J., Liu C., Ning C. (2021). Fabrication of enamel-like structure on polymer-infiltrated zirconia ceramics. Dent. Mater..

